# High-Speed Handling Robot with Bionic End-Effector for Large Glass Substrate in Clean Environment

**DOI:** 10.3390/s22010149

**Published:** 2021-12-27

**Authors:** Zhengyong Liu, Youdong Chen, Henan Song, Zhenming Xing, Hongmiao Tian, Xiaobiao Shan

**Affiliations:** 1 School of Mechanical Engineering and Automation, Beihang University, Beijing 100191, China; liuzhengyong@sineva.com.cn; 2 Hefei Sineva Intelligent Machine Co., Ltd., Hefei 230013, China; 3 State Key Laboratory of Robotics and System, Harbin Institute of Technology, Harbin 150001, China; 19B908033@stu.hit.edu.cn (H.S.); xingzm@hit.edu.cn (Z.X.); shanxiaobiao@hit.edu.cn (X.S.); 4 State Key Laboratory for Manufacturing Systems Engineering, Xi’an Jiaotong University, Xi’an 710049, China; hmtian@xjtu.edu.cn

**Keywords:** handling robot, bionic end adsorption, glass substrate

## Abstract

The development of “large display, high performance and low cost” in the FPD industry demands glass substrates to be “larger and thinner”. Therefore, the requirements of handling robots are developing in the direction of large scale, high speed, and high precision. This paper presents a novel construction of a glass substrate handling robot, which has a 2.5 m/s travelling speed. It innovatively adopts bionic end-suction technology to grasp the glass substrate more firmly. The structure design is divided into the following three parts: a travel track, a robot body, and an end-effector. The manipulator can be smoothly and rapidly extended by adjusting the transmission ratio of the reducer to 1:2:1, using only one motor to drive two sections of the arm. This robot can transfer two pieces of glass substrate at one time, and improves the working efficiency. The kinematic and dynamic models of the robot are built based on the DH coordinate. Through the positioning accuracy experiment and vibration experiment of the end-effector, it is found that the robot has high precision during handling. The robots developed in this study can be used in large-scale glass substrate handling.

## 1. Introduction

In an information-based society, display panels have become ubiquitous. With the rapid development of IT and flat panel display (FPD) industries, the market demand for display panels keeps increasing [1].

Glass substrate is a key basic material for the FPD industry [2]. The larger the glass substrate is, the greater the cutting selectivity becomes. This leads to a higher production yield and more efficient production. In the production of display panels, glass substrates need to be transferred to different environments several times for processing, including strong acidic and alkaline environments, high temperature environments, and other harsh environments [1,3]. Therefore, the handling robot not only ensures accuracy of the operation to prevent damages, but also avoids excessive harm to the operator [4,5,6,7].

**Figure 1:**
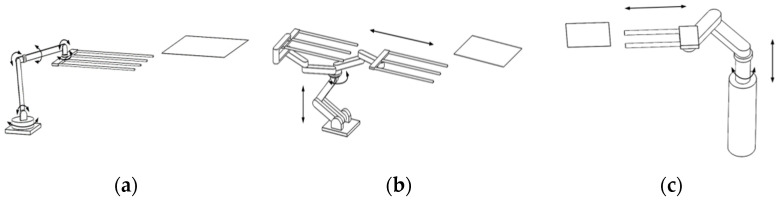
Classification of basic robot configurations by coordinate form. (**a**) Vertical multi-joint, (**b**) planar multi-joint, (**c**) cylindrical coordinate.

Currently, there are the following three configurations of glass substrate handling robots commonly used in the FPD industry, classified by coordinate forms: vertical multi-joint configuration, planar multi-joint configuration, and cylindrical coordinate configuration, as shown in Figure 1 [8]. Vertical multi-joint configuration and planar multi-joint configuration can move flexibly and have complex structures, which are suitable for small-generation FPD production lines [9]. Cylindrical coordinate configuration usually has three degrees of freedom, simple and intuitive motion, high rigidity, and mechanism precision. It can also achieve a larger working space with a more compact structure, which is suitable for high-speed, high-precision, and large-generation glass substrate handling robot design [10]. Despite all these features, the robot can only carry one glass substrate at a time [11,12,13,14].

In order to be lightweight, modular, reconfigurable, and intelligent, the robot structure and control algorithms are continuously improved and optimized through previous product designs and production practice experience [6,15]. Quite a few companies have designed and produced their own series of glass substrate handling robots [16,17], such as Yaskawa’s MOTOMAN-MFL2200 [18,19], Daihen’s SPR-8573 [20], Sankyo’s column offset configuration handling robot [21], Robostar’s AFDH1L629-545, and the cylindrical handling robot by Dalian University of Technology [22].

The grasping and transferring of large-sized glass is a very important application field in industrial production [23]. However, the transferring methods largely depend on the fragility and cleanliness of the glass. Existing methods, such as vacuum chuck and mechanical gripper, cannot effectively meet the goal of the stable transportation of large-sized glass [24,25,26,27]. This motivates a new interface suction method with strong adhesive capacity and no pollution to the surface [28,29].

This paper introduces a kind of glass substrate handling robot that meets the industry’s needs. Section 2 is the design of the robot structure, which is divided into the travel track, the robot body, and the end-effector. Section 3 is the strength verification of the structure. Section 4 is the kinematic and dynamic analysis of the robot. Section 5 is the vibration characteristics analysis of the end-effector. Finally, the conclusion is presented in Section 6.

## 2. Structure Design

**Figure 2:**
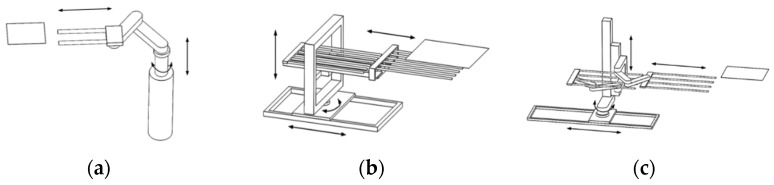
Classification of cylindrical coordinate robot configurations. (**a**) Center column configuration, (**b**) portal configuration, (**c**) column offset configuration.

**Figure 3:**
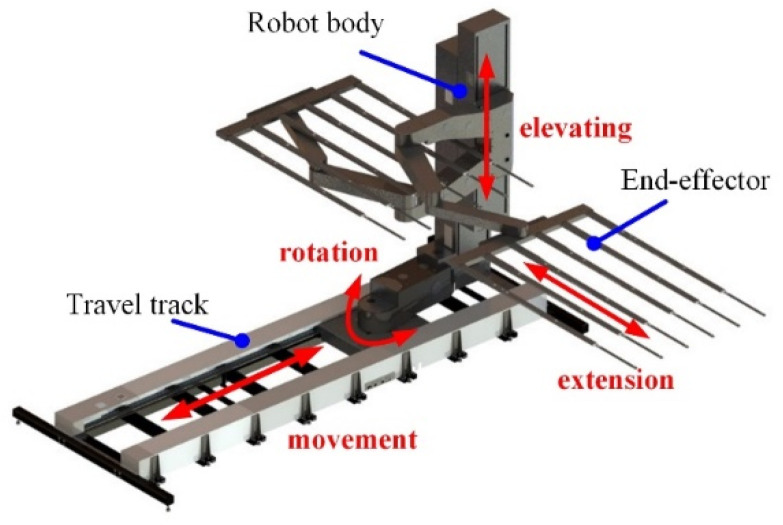
Structural configuration.

Due to the requirements of large size, high speed, and high accuracy for transferring the glass substrate, the glass substrate handling robot adopts the cylindrical coordinate configuration. The cylindrical coordinate configuration glass substrate handling robot can be subdivided into the following three configurations: the central column configuration, portal configuration, and column offset configuration, as shown in Figure 2. Among them, the cylindrical coordinate glass substrate handling robot with the column offset configuration has advantages in its size, working height, and distance—its lifting axis can be designed into multiple segments to achieve the pick-and-place of glass substrates at a high position. It is easy to implement the modular design, which can flexibly respond to different travel requirements in different transfer directions. The overall motion control of the robot is relatively simple. Therefore, the cylindrical coordinate glass substrate handling robot with column offset configuration was selected as the final design configuration. The structural configuration of the glass substrate handling robot is illustrated in Figure 3.

**Figure 4:**
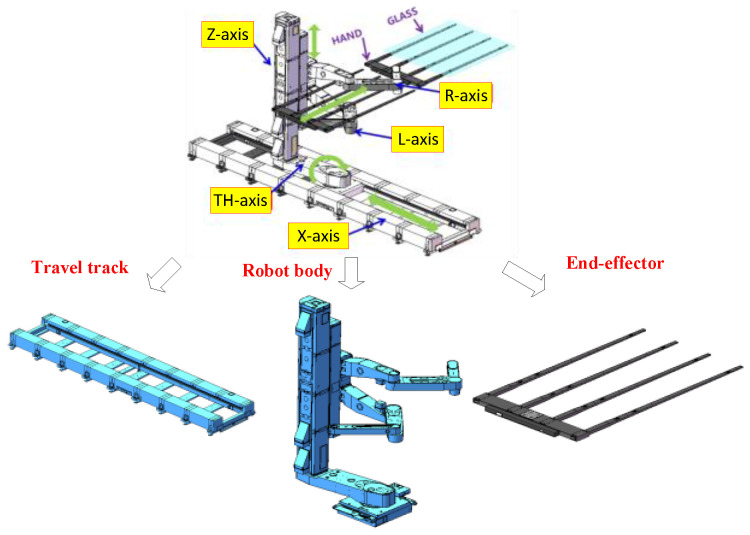
Design of the handling robot and axis system.

The robot consists of a travel track, a robot body, and an end-effector. The travel track enlarges the working space of the robot and enables the robot to move forward along the X-axis direction (see Figure 4). The end-effector is designed based on the bionic principle, and has less grasping stress and a stronger adhesion force. The robot body consists of an elevating module, a rotation module, and an arm module, which are installed on the travel track base and can adjust the space posture by rotating the TH-axis. Among them, the elevating module can move the robot along the Z-axis direction to adjust the handling height. The arm module is composed of two arms, and it can carry two glass substrates at the same time, to improve the working efficiency. Each arm has a shoulder, a forearm, and a hind arm. The joints of the arm are driven by only one drive motor. Smooth and rapid extension of the arm is realized by adjusting the transmission ratio of the reducers to 1:2:1. The end-effector is equipped with adsorption devices and sensors, and active vibration suppression is used to achieve safe and trace-free grasping of the glass substrate. The design of the mechanism of the handling robot and axis system is shown in Figure 4 and Table 1.

### 2.1. Design of the Travel Track

**Figure 5:**
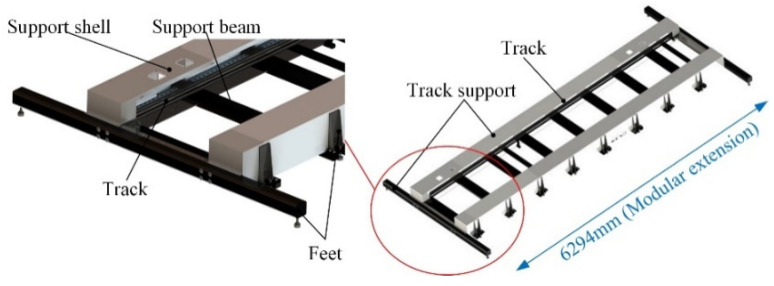
The structure of travel track.

Figure 5 shows the structure of the travel track. The travel track includes a moving track and the track support. In order to extend the movement range of the travel track, the track bracket adopts a modular design, which consists of multiple groups of modular bracket structures with the same or similar structure, and the cables are driven by the inward drag chain. The rail structure adopts the transmission method of rack and pinion drive with rail slider sliding. The modular design allows for free splicing of the moving rail and supports a 2.5 m/s moving speed.

### 2.2. Structure of the Robot Body

**Figure 6:**
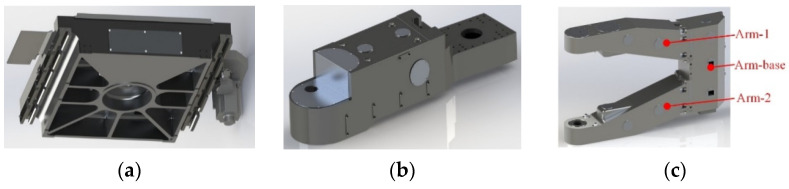
Design of handling robot. (**a**) X-axis base, (**b**) TH-axis base, (**c**) arm.

The robot body includes an elevating module, a rotation module, and both upper and lower arms. The elevating module consists of two DOFs, Z1-axis and Z2-axis. The Z2-axis and the arm support can be lifted freely. To achieve the elevating motion, the mechanical principles of the Z1-axis and Z2-axis are the same, using the ball screw drive with rail slider sliding [29]. The two arm bases are equipped with drive motors and the output shafts are connected to the output of the reducer of the arm mechanism, and the upper and lower arm mechanisms are arranged symmetrically with the same design. The motion of the arm mechanism is only driven by a driving motor in the arm base, and the transmission ratio of the reducer at each joint is adjusted to 1:2:1 to achieve the smooth translational motion of the arm. The two arms can grasp two pieces of glass substrate at one time. In order to ensure that the handling robot is as lightweight as possible, the stiffness of its shell structure is optimized, as shown in Figure 6.

### 2.3. Design of the End-Effector

**Figure 7:**
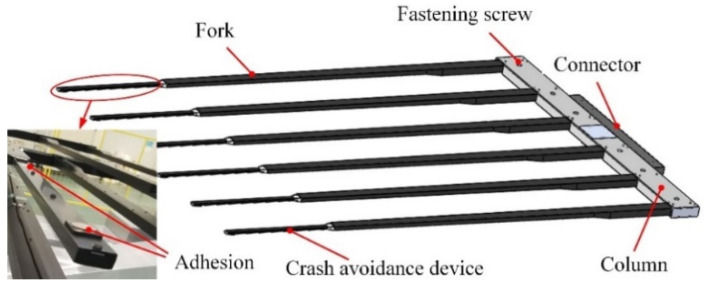
The structure of the end-effector.

The end-effector is designed to be a cantilever structure. Multiple forks are designed to enlarge the contact area with the glass substrate, with dimensions of 2940 mm × 3370 mm × 0.5 mm. To meet the rigidity requirements, the structural mass is significantly reduced by using carbon fiber in the end-effector, and using an aluminum alloy in the connection and fasteners. The product structure is shown in Figure 7.

Adhesion is the key to grasp the glass substrate while transferring it. This paper uses dry adhesion to achieve the pick-and-place motion. Compared with other adhesive mechanisms, dry adhesion and its functionalized artificial surface, relying solely on intermolecular forces, have the following advantages: (1) Since there is no need to introduce chemical binders or other liquid materials into adhesion or desorption processes, they do not cause material retention on the surface, which is undoubtedly the best option for the adhesion process with cleanliness requirements. (2) There is no obvious time delay for the occurrence and disappearance of adhesion, which is suitable for rapid grasping and release. (3) There is no need to use field assistance (thermal, magnetic, or electrical). Thus, it is convenient for miniaturization, integration, and flexible operation. (4) It is not sensitive to environmental parameters, such as temperature, humidity, and vacuum. Thus, it is suitable for adhesion operations in complex and changeable, or even extreme, environments. In view of these advantages, in recent years, dry adhesive functional surfaces imitating the complex micro–nano structure of gecko soles have been widely used in many fields, such as medical and health, bionic robots, picking devices, or manipulators [30].

The gecko, a typical climbing animal, displays excellent crawling ability in different environments and on different surfaces. It has provided a lot of inspiration for the design and manufacturing of interface adhesive materials [31]. The surface of the gecko’s sole has a typical hierarchical microstructure. Each toe has a millimeter-scale oblique flake-shaped structure, which is called a lamella, and each lamella grows a micron-scale seta array, and each seta is divided into hundreds of nano-scale spatula-type contact units at its end. This highly discretized structural feature of the end can achieve effective contact with various surfaces, and effectively inhibit crack propagation at the interface. It was found that van der Waals forces are the main source of gecko adhesion. Since it only relies on intermolecular force, without any chemical adhesives, it is also known as dry adhesion.

**Figure 8:**
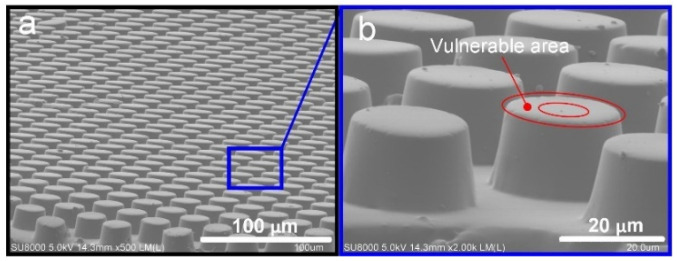
The flexible cylindrical structures. (**a**) the SEM picture of large-area cylindrical structures; (**b**) the illustration of vulnerable area of cylindrical structure.

In this study, the working conditions require ultra-clean, low vacuum, microgravity, or ultra-low contact stress, and the bionic dry adhesion effect is irreplaceable when attempting to achieve smooth functions, such as transferring, attaching, and grasping. The mushroom-like structure, with a flat disc at the end of the gecko’s sole, has the most significant adhesion. The introduction of the end disc increases the actual contact area of the structure, and the mechanism of adhesion enhancement is closely related to the complex interface physical behavior and debonding mode [32]. Generally speaking, the debonding behavior of flexible cylindrical structures is often controlled by crack nucleation and propagation in the contact edge, as shown in Figure 8. Under tensile force, the cracks are very easily eroded into the inside, and eventually cause destruction of the entire contact interface and adhesion failure.

**Figure 9:**
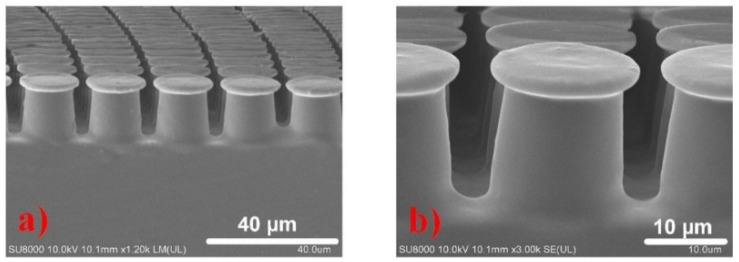
(**a**). SEM image of the large-sized fabricated artificial mushroom-shaped dry adhesion structure. (**b**). The close-up of the single mushroom-shaped structure, which has a height of 20 μm, bottom pillar diameter of 16 μm, and top cap of 18 μm.

The “column–surface” contact is replaced with the “plate–surface” contact in this study, as shown in Figure 9. The external load does not directly act on the disk part outside the column by stretching or squeezing. Therefore, the contact stress on the outside of the column is smaller than the contact stress on the inside. Based on the stress distribution, the cracks are difficult to erode into the internal area, thus ensuring the integrity of the contact interface under the cylinder. In addition, the mushroom-shaped structure flattens the stress in the contact area and greatly reduces the stress concentration at the edge, making it easier for cracks to nucleate inside the contact interface under tensile load, and the higher the strength of the interface is, the stronger the adhesion force becomes.

**Figure 10:**
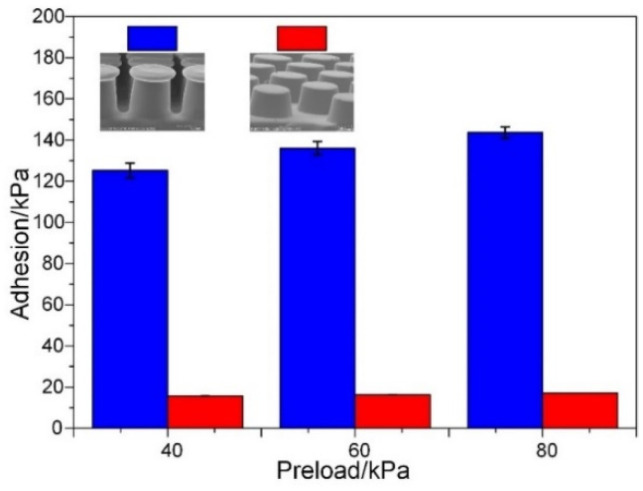
Comparison of adhesion between the artificial mushroom-shaped dry adhesion structure and cylindrical structures of the same size.

Figure 10 shows the comparison of adhesion between the artificial mushroom-shaped dry adhesion structures and cylindrical structures of the same size. Clearly, the adhesion of the mushroom-shaped dry adhesion structures is much larger than the adhesion of the cylindrical structures. Under 80 kPa preload, the saturated adhesion of the mushroom-shaped dry adhesion structures is about 8.5 times more than the saturated adhesion of the cylindrical structures. Additionally, the mushroom-shaped dry adhesion structures reach 125 kPa adhesion under 40 kPa preload. The ability of the mushroom-shaped dry adhesion structures to achieve a high adhesion strength with a relatively low preload is a huge advantage, and is desirable for the transportation of fragile objects.

**Figure 11:**
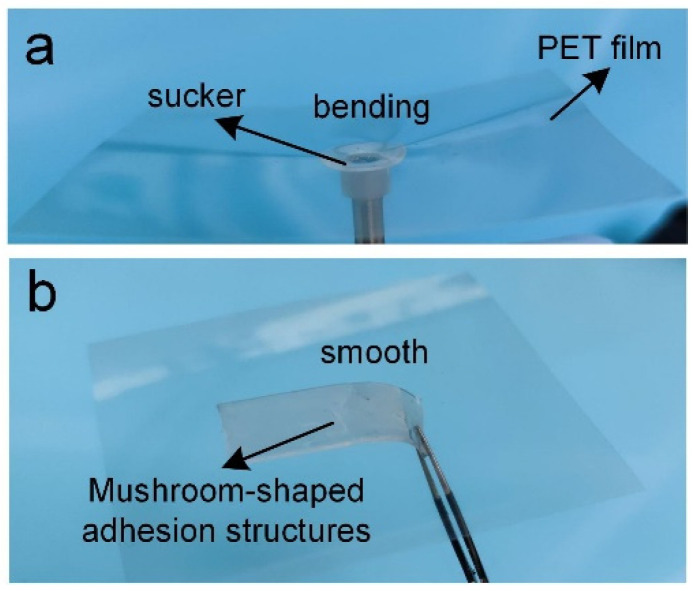
Comparison of using commercial suction cup and adhesive structures to pick up PET film. (**a**) the PET film bending when using a commercial suction cup to pick it up; (**b**) the mushroom-shaped dry adhesion structure making the PET film flat and smooth when picking it up.

Because the mushroom-shaped dry adhesion structure distributes the stress over a larger contact area, and greatly reduces the stress concentration at the edge, it does not damage the objects when transferring ultra-thin objects. As shown in Figure 11, the PET film bends because of the stress concentration when using a commercial suction cup to pick it up. However, the mushroom-shaped dry adhesion structure could make the PET film flat and smooth when picking it up.

## 3. Structural Strength Verification

Since the handling robot was designed with the characteristics of high precision and light weight, as listed in Table 2, it is necessary to use finite element analysis to perform a static and modal analysis on the key load-bearing components of the robot. This is an essential step to evaluate the structural strength of the handling robot, and to verify the design feasibility.

**Figure 12:**
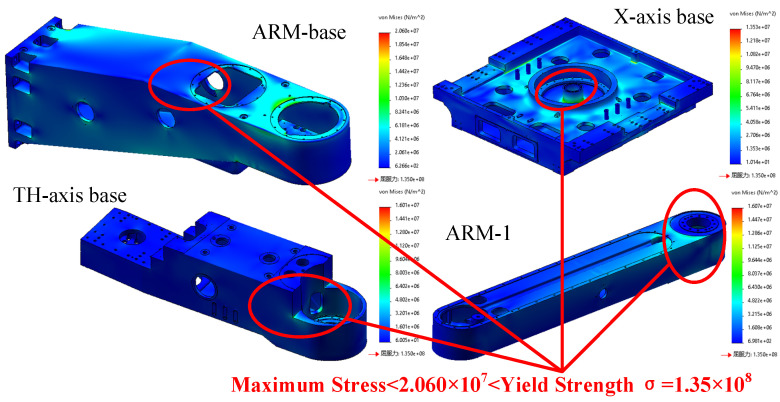
Stress cloud.

**Figure 13:**
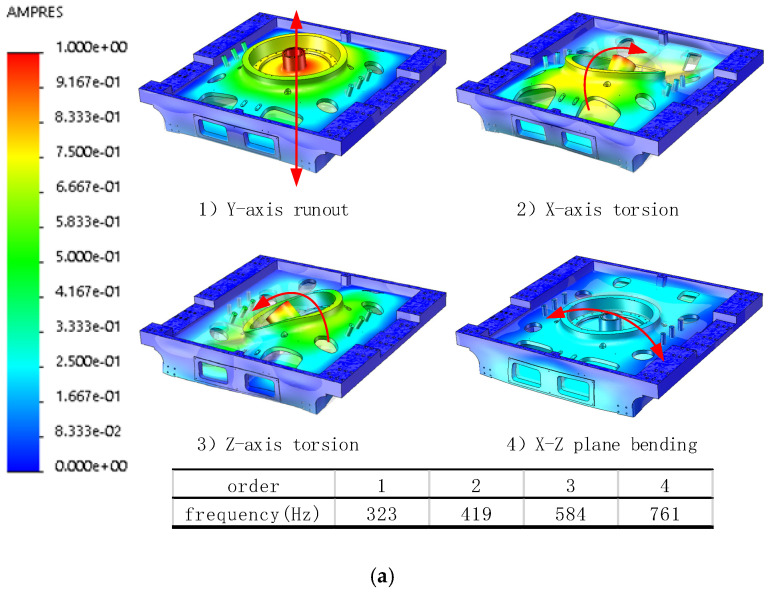

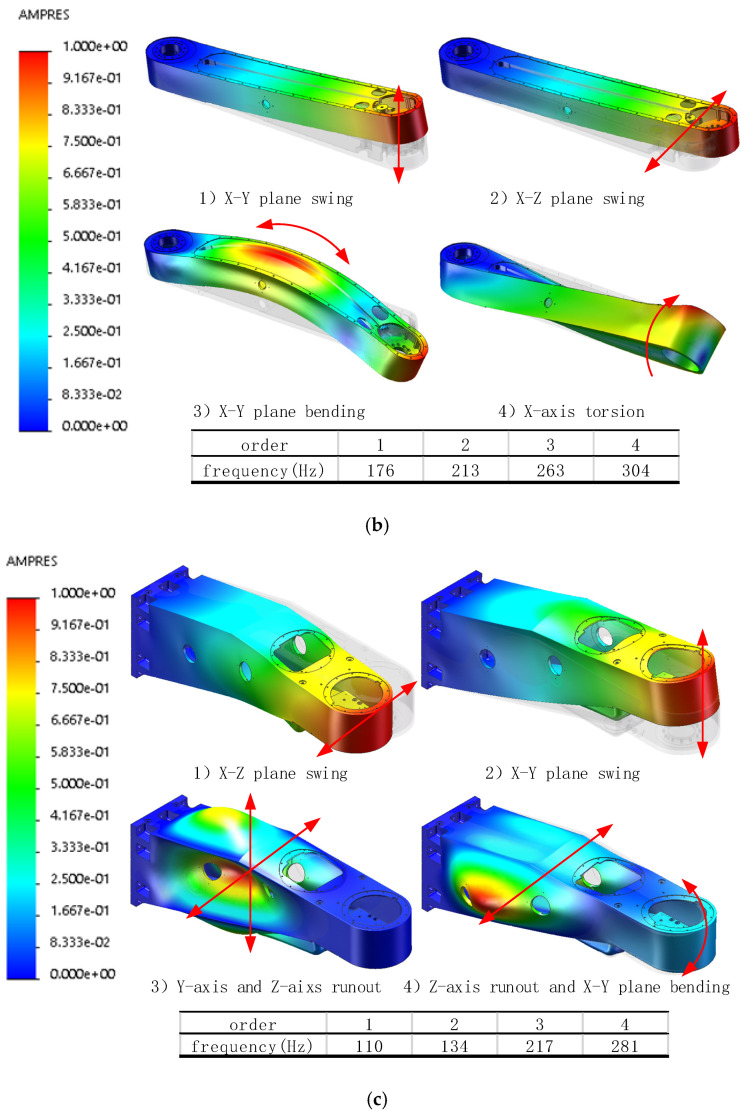

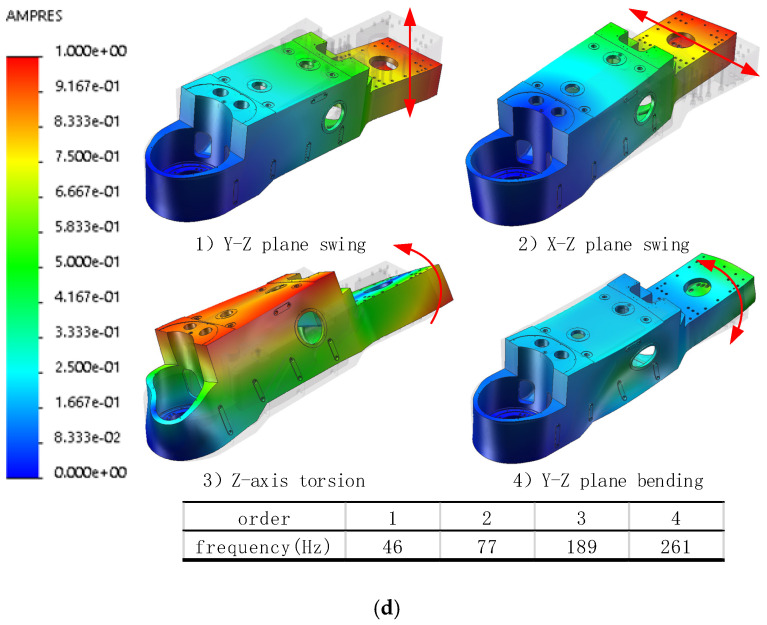
Finite element simulation: (**a**) X-axis base modal cloud, (**b**) ARM-1 modal cloud, (**c**) ARM base modal cloud, (**d**) TH-axis base modal cloud.

Using Cosmos Works finite element analysis module, the strength of the key components of the handling robot was checked. There are four structures in this robot that carry out the statics and modal simulation analysis. The stress cloud chart is shown in Figure 12, and the modal clouds are shown in Figure 13. This paper obtains the modal simulation results on an equal basis, through normalized data. The maximum equivalent stress is much less than the yield strength of the corresponding casting aluminum alloy material. The natural frequency is compared. This modeling study validates that the handling robot has good structural strength and dynamic performance, and the design feasibility is met.

## 4. Robot Modelling and Control

### 4.1. Kinematic Analysis

**Figure 14:**
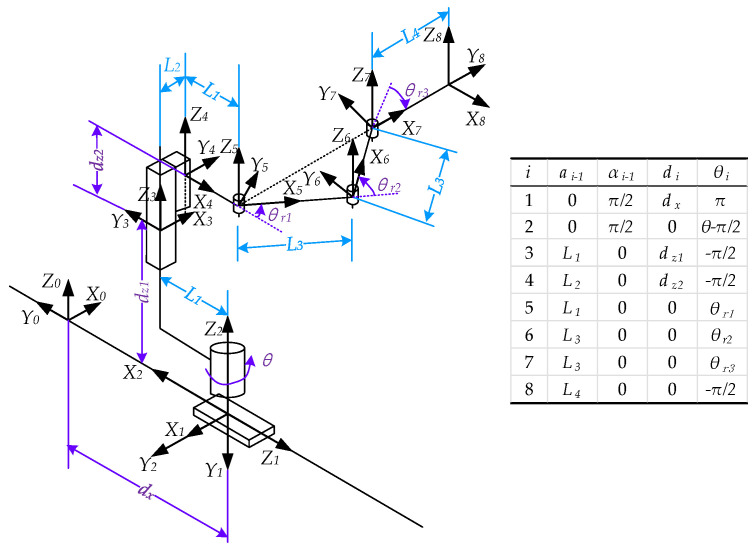
Mechanism sketch, coordinate systems and DH parameter table of the handling robot.

Since the handling robot is relatively simple in structure, kinematic analysis is carried out by the geometric method and the corresponding coordinate transformation method. The mechanism sketch, DH coordinate system, whose coordinate system 8 is established on the tip of the fork, and its DH parameter table are shown in Figure 14.

According to DH method, the forward kinematics formula is as follows,

(1) T80=[c(θ+θr1+θr2+θr3)−s(θ+θr1+θr2+θr3)0pxs(θ+θr1+θr2+θr3)c(θ+θr1+θr2+θr3)0py001pz0001]

where

$$\begin{array}{l} p_{x}=L_{2}c\theta +L_{3}\left(s\left(\theta +\theta_{r1}\right)+s\left(\theta +\theta_{r1}+\theta_{r2}\right)\right)+L_{4}s\left(\theta +\theta_{r1}+\theta_{r2}+\theta_{r3}\right),\\ p_{y}=L_{2}s\theta -L_{3}\left(c\left(\theta +\theta_{r1}\right)-c\left(\theta +\theta_{r1}+\theta_{r2}\right)\right)-L_{4}c\left(\theta +\theta_{r1}+\theta_{r2}+\theta_{r3}\right)-d_{x},\\ p_{z}=d_{z1}+d_{z2}. \end{array}$$

$L_{1}$, $L_{2}$, $L_{3}$ and $L_{4}$ are dimension parameters, as shown in Figure 14. $d_{x}$, θ, $d_{z1}$, $d_{z2}$, $\theta_{r1}$, $\theta_{r2}$ and $\theta_{r3}$ are the joint variables of the robot. s, c, etc. are the simple expressions of trigonometric functions; c is for cos and s is for sin.

Assume that $O_{i}$ is the origin of coordinate system i. According to the mechanical constraint of the robot arm, i.e., the fork always moves along the line from $O_{5}$ to $O_{7}$ and $O_{5}O_{6}=O_{6}O_{7}$, the following relationship is derived.

(2) $$\begin{array}{l} \theta_{r2}=\pi -2\cdot \theta_{r1}\\ \theta_{r3}=\theta_{r1}-\pi /2 \end{array}$$

Additionally, the forward kinematics formula of the handling robot can be derived as follows:

(3) T80=[−sθ−cθ0(L2+L4+2L3sθr1)cθcθ−sθ0(L2+L4+2L3sθr1)sθ−dx001dz1+dz20001]

### 4.2. Dynamic Analysis

The Lagrange method is used in this paper to build the dynamics equations of the handling robot and analyze its dynamics characteristics. The Lagrange function *L* is defined as follows:

(4) L=K−P

where K and P are the kinetic energy and potential energy, respectively. We define the generalized coordinates $q=\left(d_{x},\theta ,d_{z1},d_{z2},\theta_{r1},\theta_{r2},\theta_{r3}\right)$, so the dynamic equation described by the Lagrange function *L* is as follows:

(5) $$T_{i}=\frac{d}{dt}\left[\frac{\partial L}{\partial {\dot{q}}_{i}}\right]-\frac{\partial L}{\partial q_{i}}$$

where i=1,2,…,6,7 is the number of each joint of the handling robot, ${\dot{q}}_{i}$ is the generalized velocity, and $T_{i}$ is the generalized force.

Generally, there are two ways to obtain the robot inertial parameters: one is identification methods [33], and the other is CAD measurement. Since we have the 3D model of the robot, we obtain the joint mass and inertia parameters of the handling robot through CAD measurement, as shown in Table 3.

Considering the effect of frictional force on the handling robot, the robot dynamics equation is as follows:

(6) $$T=M\left(q\right)\ddot{q}+C\left(q,\dot{q}\right)\dot{q}+g\left(q\right)+\tau_{f}$$

where *M*(*q*) is the inertia term, $C\left(q,\dot{q}\right)\dot{q}$ is the centrifugal force and Coriolis force term, *g*(*p*) is the gravity term, and $\tau_{f}$ is the friction term. We choose Coulomb friction and viscous friction model in this paper, which is $\tau_{f}=fv\cdot \dot{q}+fc\cdot sign\left(\dot{q}\right)$, where fv and fc are the diagonal matrices of the viscous and Coulomb friction parameters, respectively.

### 4.3. Robot Control System

#### 4.3.1. Hardware Level

In the actuator system of the handling robot, each axis is driven by a motor. By building the 3D model of the whole robot, the load of each joint is obtained, and then dynamic simulation can be executed, which guides the motor and driving element selection. Among them, the X-axis is driven by a motor and gear-rack with rail-slider to realize fast and stable movement; the Z1-axis and Z2-axis are driven by a motor and screw with rail-slider to realize lifting movement; and the TH-axis is driven by motor to realize rotary motion. Both the R-axis and L-axis are driven by one motor with two groups of pulleys and a reducer to realize the horizontal and stable movement of the end-effector.

**Figure 15:**
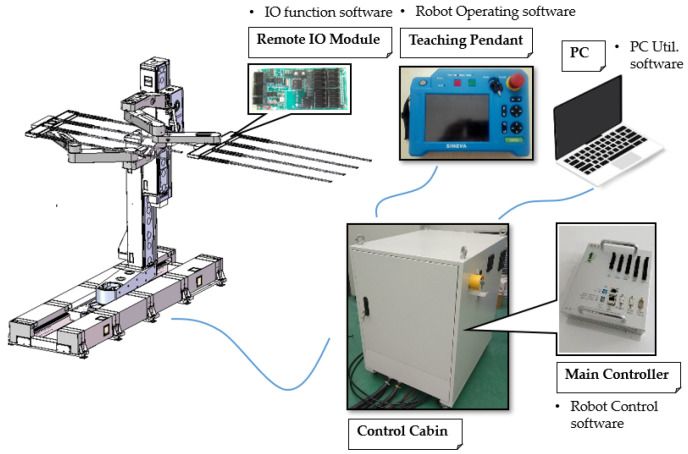
The control system of the handling robot.

With the above contents, the control system is designed. The control system of the robot is shown in Figure 15. Robot application program is developed in the PC and is sent to the main controller via ethernet. The main controller executes the robot application program and sends control commands to the servo drivers in the control cabin. Then, the servo drivers control the motors by the control commands and read the feedback of the encoders in the motors. In addition, the remote IO module communicates with the main controller by the robot input/output. Moreover, a teaching pendant is used to control the robot in manual mode. An alternating current source is used to provide power to the control cabin. Among them, the servo drivers and motors adopt commercially available mature products. The main controller is independently designed. The main processor of the main controller connects each peripheral chip or module through a Group Policy Management Console (GPMC) bus, including an NAND FLASH chip, Magnetoresistive Random Access Memory (MRAM) chip, Field Programmable Gate Array (FPGA) chip, Ethernet interface expansion chip, Controller Area Network (CAN) bus expansion chip and Ethernet Control Automation Technology (EtherCAT) module.

Several sensors are also applied in the handling robot. Among them, each motor is equipped with an encoder, and the integration of electrical components is equipped with a temperature protector. Photoelectric switches are applied to the end effector to detect the position and movement state of the glass substrate. Length sensors are applied on the arm to ensure that there are no obstacles in the workspace, and proximity sensors are used to cooperate with the motors to find zero. Photoelectric switches are used to detect the motion state on the X-axis, TH-axis and Z-axis. Through the cooperation of the above sensors, the accurate, stable and safe operation of the handling robot is ensured.

#### 4.3.2. Software Level

In order to ensure that the robot tracks the desired trajectories, the robot controller algorithm is designed. In this study, the classical computed torque method [34] is applied to the trajectory tracking control. By assuming that the desired joint trajectory $q_{d}\left(t\right)$ is the function of time, the desired joint position $q_{d}$, desired joint velocity ${\dot{q}}_{d}$, and desired joint acceleration ${\ddot{q}}_{d}$ can then be output by a trajectory planning algorithm at any moment [35]. Afterwards, the model-based control law and servo control law of the robot can be designed, as follows:

(7) $$\tau \;=\;\alpha \tau^{\prime }+\beta$$

(8) $$\tau^{\prime }\;=\;k_{p}e+k_{v}\dot{e}+{\ddot{q}}_{d}$$

where $\tau \in {ℜ}^{n\times 1}$ denotes the model-based control law, $\alpha \in {ℜ}^{n\times n}$ is the gain matrix, $\beta \in {ℜ}^{n\times 1}$ is the nonlinear function vector used to linearize the robot system, $\tau^{\prime }\in {ℜ}^{n\times 1}$ is the servo control law, $e,\dot{e}\in {ℜ}^{n\times 1}$ are the joint position error and the joint velocity error, respectively, $k_{p}$ is the joint position error gain, and $k_{v}$ is the joint velocity error gain.

Then, by defining α=M(q) and $\beta =C\left(q,\dot{q}\right)\dot{q}+g\left(q\right)+\tau_{f}$, the control law above can become the following:

(9) $$\tau =M\left(q\right)\left(k_{p}e+k_{v}\dot{e}+{\ddot{q}}_{d}\right)+C\left(q,\dot{q}\right)\dot{q}+g\left(q\right)+\tau_{f}$$

If the model-based control law is imposed on the robot object, namely, letting $\tau =T_{i}$, the following equation can be easily derived:

(10) $$\ddot{q}=k_{p}e+k_{v}\dot{e}+{\ddot{q}}_{d}$$

By defining the joint acceleration error $\ddot{e}={\ddot{q}}_{d}-\ddot{q}$, the error space equation of the robot control system can be obtained as follows:

(11) $$\ddot{e}+k_{v}\dot{e}+k_{p}e=0$$

Depending on the equation above, the desired response of the robot control system can be designed by selecting appropriate gain coefficients. When the dynamic model of the robot is more accurate, and there is less measurement/vibration noise or fewer initial errors, the performance of robot trajectory tracking will be better. In fact, Equation (11) sets a balance among accuracy in tracking and dynamic response. This is not only a problem of knowing the exact model but also that of the feasibility of the trajectory within a given bandwidth. However, in the actual robot control system, it is difficult to know the accurate dynamic model of the robot, due to the existence of measurement/vibration noise and initial errors. Therefore, tracking errors inevitably exist. In addition to the above, there are many more issues that can affect the tracking errors, as follows: 

(a) Variable and not constant friction.

(b) Internal resonant frequencies (the links can act as elastic bodies).

(c) Saturation on motor, currents and drivers.

(d) Delays in control loops.

(e) Sensor placement, accuracy and bandwidth.

## 5. Experiment

The end-effector of the glass substrate handling robot is the mechanism that has the closest contact between the handling robot and the glass substrate. Due to the requirement of meeting the design characteristics of high speed and light weight, the main body of the handling robot inevitably generates vibration excitation to the end-effector during operation. As the glass substrate is thin, and has a large size and high brittleness, slight vibration tends to break the glass substrate when it is in contact with the end-effector. Therefore, this paper introduces an end-effector for the glass substrate handling robot and analyzes its vibration characteristics.

### 5.1. Trace Experiment

Since the high-speed and lightweight robot will inevitably cause vibration of the end-effector in practical applications, a piezoelectric element is set on the end-effector to reduce its vibration. The piezoelectric element is fit into an actuator that suppresses the vibration by controlling the piezoelectric signal acting on the piezoelectric sheet. A trace experiment of the end-effector is carried out to measure the position error and verify the vibration suppression effect.

**Figure 16:**
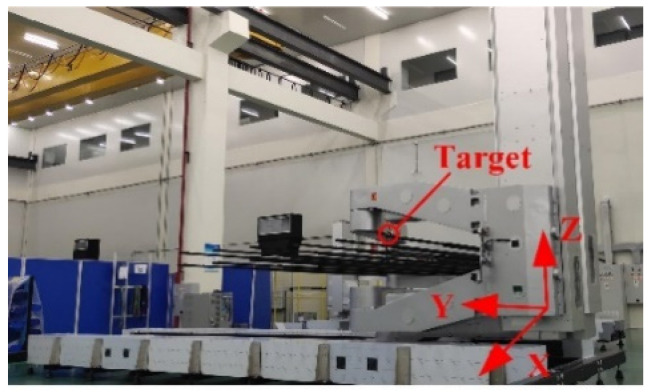
Experimental diagram.

The target of the FARO laser tracker was installed at the end of the end-effector. The end-effector moves forwards and backwards to collect the displacement of the target ball, as shown in Figure 16.

**Figure 17:**
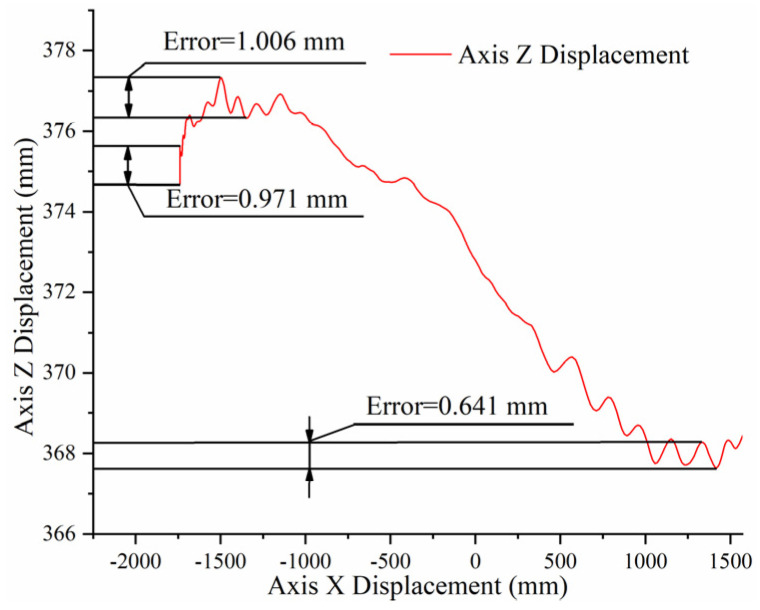
Positioning error graph.

As shown in Figure 17, the curve is the track in the Z-axis direction, when the end-effector is contracted. The obvious undulation of the curve is explained by the fact that the end-effector has an upward deflection at the beginning of its design. Therefore, with the contraction of the end-effector, the target forms an action curve with an initial increase followed by a continued decrease, which is consistent with the overall trend of the acquisition curve. Although there was a difference between the adjacent points of the curve, the max position error was within the range of 1.006 mm. This shows that the robot has high precision.

**Figure 18:**
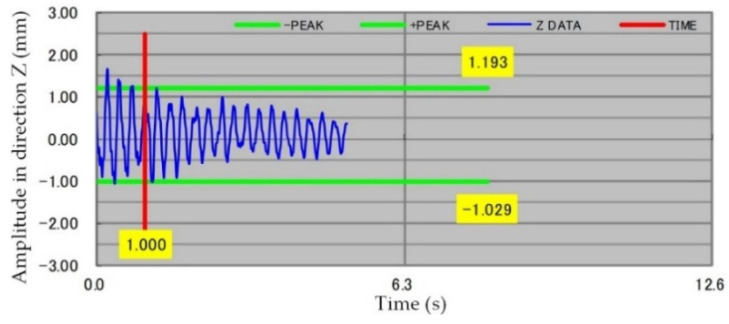
The vibration amplitude of the Z-axis.

The end-effector moves to collect the displacement of the target ball in the Z direction. The vibration amplitude is shown in Figure 18.

As shown above, the amplitude of the vibration of the end-effector is less than 2 mm. In actual handling, the damage rate of the glass substrate is low, indicating that the handling robot can safely pick up and place the glass substrate.

### 5.2. Fork Vibration Characteristic Experiment

**Figure 19:**
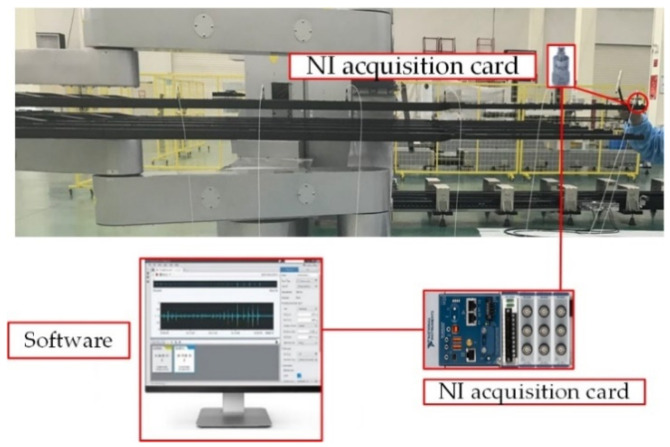
The experimental setup.

**Figure 20:**
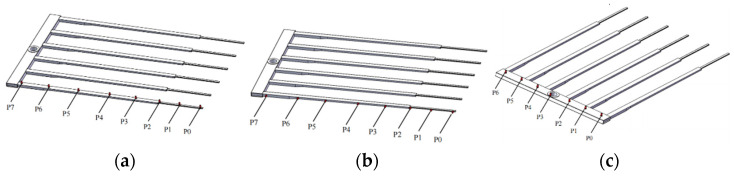
The different installation positions of sensors. (**a**) The sensors were installed on a single fork in the Z-axis direction; (**b**) The sensors were installed on a single fork in the Y-axis direction; (**c**) The sensors were installed on the connector of the fork.

**Figure 21:**
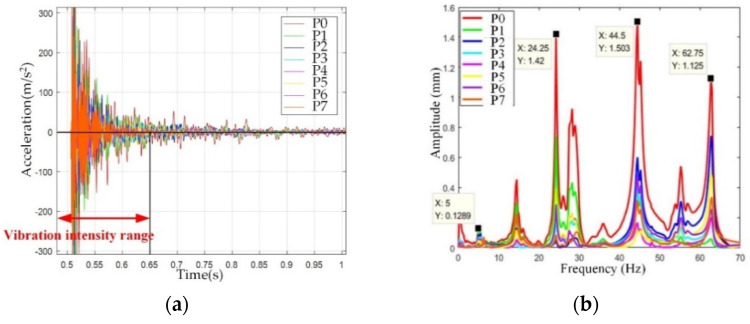
Fork Z-direction vibration mode. (**a**) Time domain response diagram, (**b**) frequency domain response diagram.

**Figure 22:**
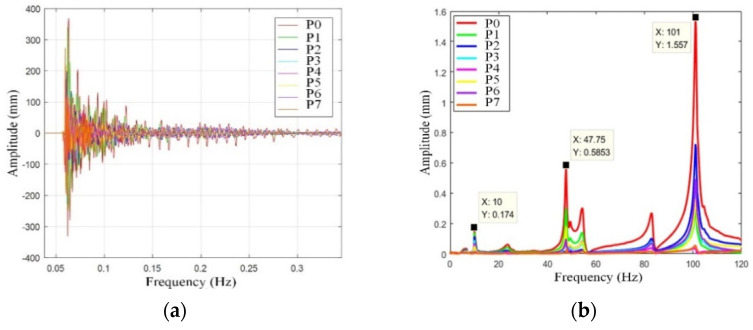
Fork Y-direction vibration mode. (**a**) Time domain response diagram, (**b**) frequency domain response diagram.

**Figure 23:**
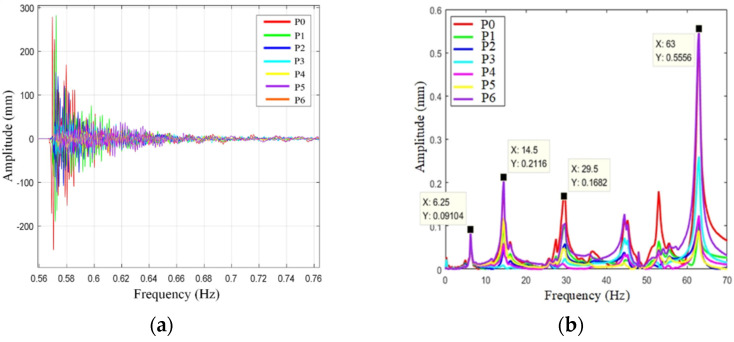
Column-hand Z-direction vibration mode. (**a**) Time domain response diagram, (**b**) frequency domain response diagram.

To gain a better understanding of fork vibration characteristics, the fork vibration modal test is carried out. The experimental setup is shown in Figure 19, where the software and hardware used in the experiment and the experimental topology are illustrated. The sampling frequency of the experiment is 8192 Hz, and the number of sampling points is 30,000. The acceleration sensors are fixed at point 0–7 on the fork of the end-effector, as shown in Figure 20a,b. In order to measure the natural frequency of the structure, the method of force shock excitation is adopted. A hammer was used to tap the measurement points to excite the fork into a vibration state, and the vibration characteristics can be revealed by the experiment data. Because the vibration of the end-effector is poly-directional, different installation positions are used to judge whether different excitation points have an impact on the natural frequency of the system, as shown in Figure 20. The sensors were installed on a single fork in the Z- and Y-axis directions, respectively, as shown in Figure 20a,b. Another installation position is on the connector of the fork, as shown in Figure 20c. The time domain and the frequency domain responses are shown in Figure 21, Figure 22 and Figure 23.

Figure 21 shows the amplitude of 0–7 points at the Fork in the Z direction. As can be observed in Figure 21b, the first-order natural frequency of the fork in the Z direction is 5 Hz, and the second-order natural frequency of the fork in the Z direction is 24.25 Hz. Between the first-order natural frequency and the second-order natural frequency, there is a peak at 14.5 Hz in the frequency domain response diagram in Figure 21. By phase analysis of the frequency response, and the subsequent column-hand modal analysis results, the peak at 14.5 Hz is in fact a rigid vibration mode caused by the column hand’s natural vibration. Figure 21a shows that the end position of the end-effector has the largest vibration amplitude, hence the glass substrate is prone to being damaged at the end during handling. However, the first- and second-order natural frequency of the fork in the Z direction are far from the natural frequency of the glass substrate at about 74 Hz, which gives a guarantee of the safe transfer of glass substrates.

Figure 22 shows the amplitude of 0–7 points at the Fork in the Y direction. The first-order natural frequency of the fork in the Y direction is 10 Hz, and the second-order natural frequency of the fork in the Y direction is 47.75 Hz. Each of the two frequencies are still less than the natural frequency of the glass substrate at 74 Hz, which also ensures the safe transfer of glass substrates.

Figure 23 shows the amplitude of 0–6 points at the column hand in the Z direction. The first-order natural frequency of the column hand in the Z direction is 6.25 Hz, and the second-order natural frequency of the column hand in the Z direction is 14.5 Hz. Each of the two frequencies are less than the natural frequency of the glass substrate.

In summary, the first- and second-order natural frequencies of the fork in the end-effector in the Y and Z directions, and the column hand in the Z direction, are much less than the natural frequency of the glass substrate. Thus, mechanical resonance barely occurs in practice.

### 5.3. Robot Functional Test

**Figure 24:**
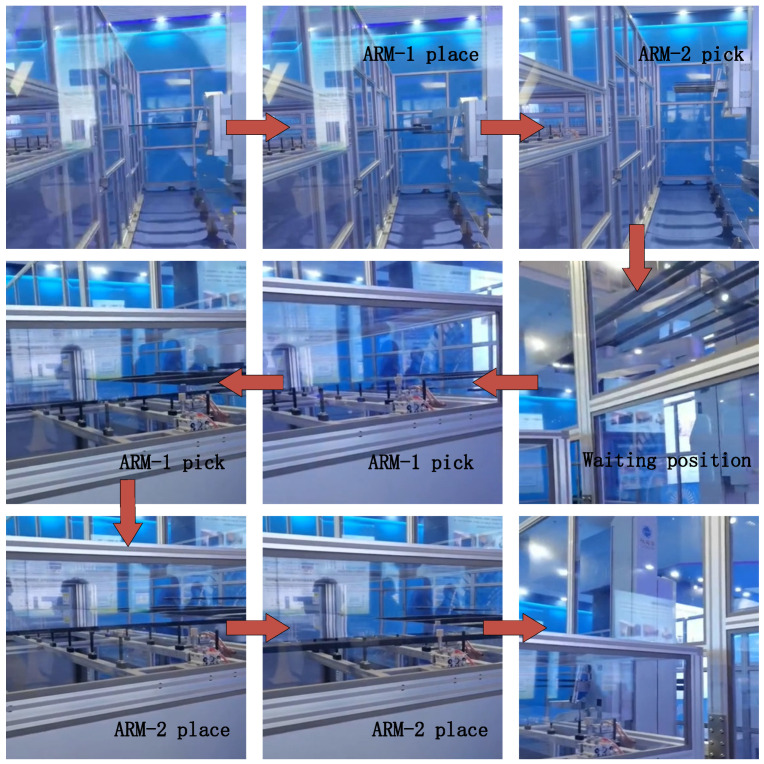
Functional test of robot.

The overall function of the glass substrate handling robot was finally tested, as shown in Figure 24. The robot places the glass substrate on ARM-1at station 1 and picks up the glass substrate on station 1 with ARM-2, and then the glass substrate is transferred to a waiting position through movement of the travel track, rotary axis, and elevating axis. Next, after receiving the command, the robot picks up the glass substrate on station 2 with ARM-1 and places the glass substrate on station 2 with ARM-2. In the test, the glass substrate handling robot picks up and places the glass substrate safely, quickly, and accurately, which meets the transportation function requirements of high-speed and high-precision large-generation glass substrate handling.

## 6. Conclusions

In this paper, a large-scale, highly clean glass substrate handling robot was designed and manufactured.

(a) Only five motors drive the robot to perform six groups of actions and transfer two glass substrates at one time, which greatly increased the work efficiency. The reduction in the motor and efficient design of the structure meets the lightweight and high stiffness design requirements.

(b) The structure of the end-effector is created to mimic the adsorption element of a bionic structure. A new dislocation-free and controllable grab method is created during handling. The proposed adsorption structure avoids the pneumatic device of the vacuum adsorption structure and makes the end actuator more lightweight. At the same time, the glass damage caused by the failure of the adsorption structure is reduced.

(c) Through the experiment of the vibration characteristics of the end-effector, it is found that the robot has high precision and hardly damages the glass substrate during handling.

Therefore, the glass substrate handling robot in this study is a significant development in the large-scale, high-speed and high-precision robot field.

## Figures and Tables

**Table 1:** Robot axis system design.

No.	1	2	3	4	5
Axis	X-axis	TH-axis	Z-axis	R-axis	L-axis
Description	travel track	Rotary axis	elevating axis, consisting of two sections of Z1-axis and Z2-axis when the stroke is larger	Upper arm movement axis	Lower arm movement axis

**Table 2:** Parameters of handling robot.

Characteristics	Value
Arm load	≥65 kg
Maximum travel speed	≥2.5 m/s
Repeatability	±0.25 mm
Transverse vibration	≤7 mm
Glass substrate size	≥2940 mm × 3370 mm
Glass substrate thickness	Minimum 0.5 mm
Environmental cleanliness level	Better than Class10 (0.3 μm)

**Table 3:** Joint mass and inertia parameters of the handling robot.

i	m/kg	$I/kg\cdot m^{2}$
1	587.52	$\left[\begin{array}{ccc} 67.80 & -1.94 & 5.38\times 10^{-2}\\ -1.94 & 72.41 & -1.38\\ 5.38\times 10^{-2} & -1.38 & 134.54 \end{array}\right]$
2	1235.88	$\left[\begin{array}{ccc} 1960.46 & 73.38 & -594.77\\ 73.38 & 2579.15 & -65.73\\ -594.77 & -65.73 & 736.52 \end{array}\right]$
3	615.37	$\left[\begin{array}{ccc} 653.17 & -3.84\times 10^{-1} & 4.41\times 10^{-1}\\ -3.84\times 10^{-1} & 664.96 & 17.08\\ 4.41\times 10^{-1} & 17.08 & 43.91 \end{array}\right]$
4	400.88	$\left[\begin{array}{ccc} 110.77 & 12.12 & 18.48\\ 12.12 & 314.15 & 4.70\times 10^{-1}\\ 18.48 & 4.70\times 10^{-1} & 221.10 \end{array}\right]$
5	151.29	$\left[\begin{array}{ccc} 46.30 & 25.21 & -1.80\\ 25.21 & 16.93 & -3.13\\ -1.80 & -3.13 & 61.27 \end{array}\right]$
6	68.48	$\left[\begin{array}{ccc} 17.76 & -9.83 & 3.26\times 10^{-1}\\ -9.83 & 6.32 & -5.67\times 10^{-1}\\ 3.26\times 10^{-1} & -5.67\times 10^{-1} & 23.58 \end{array}\right]$
7	75.58	$\left[\begin{array}{ccc} 104.90 & 4.54\times 10^{-2} & 7.08\times 10^{-4}\\ 4.54\times 10^{-2} & 49.10 & -3.48\times 10^{-1}\\ 7.08\times 10^{-4} & -3.48\times 10^{-1} & 153.88 \end{array}\right]$

where *m* is the mass of each joint of the handling robot, and *I* is the inertia matrix of each joint with centroid as reference.
